# DOES DEPRESSION IMPROVE DURING RETIREMENT AMONG BINGE DRINKERS?

**DOI:** 10.1093/geroni/igac059.2141

**Published:** 2022-12-20

**Authors:** Antonia Diaz-Valdes, Christina Sellers, Julian Ponce

**Affiliations:** Universidad Mayor, Las Condes, Region Metropolitana, Chile; Simmons University, Boston, Massachusetts, United States; University of California, Berkeley, California, United States

## Abstract

Data from the Health and Retirement Study (1992-2016) was used. All non-institutionalized respondents aged 50+ were included in our sample (n=12,618). Mixed models were conducted to study the association between transitioning to retirement and depressive symptoms (CESD) among retirees, and to test the mediation effect of alcohol use.

Results: Those retired for at least 6 years had increased probability of binge drinking. Additionally, binge drinking mediated the association between the retirement transition and depressive symptoms, making the effect stronger for those retired for 3 to 5 years (p< 0.05), and weaker and no significant for those retired for 6+ years (p>0.05). Thus, the decrease on depressive symptoms was higher for those retired between 3 to 5 years when being binge drinkers. Discussion and Implications: Addressing depressive symptoms and binge drinking among older adults is sorely needed. Binge drinking was associated with decreased depressive symptoms, consistent with the self-medication hypothesis. Treatment and screening for depression as well as binge drinking are especially important as both are associated with increased mortality. Treating depression would reduce the risk of increased alcohol use and its detrimental effects on health.

